# Strengthening RSV prevention in early life through new generation strategies*

**DOI:** 10.1016/j.jped.2025.101445

**Published:** 2025-09-19

**Authors:** Asuncion Mejias, Octavio Ramilo

**Affiliations:** St. Jude Children’s Research Hospital, Department of Infectious Diseases, Memphis, TN, USA

Respiratory syncytial virus (RSV) remains a leading cause of viral lower respiratory tract infection (LRTI, including bronchiolitis and pneumonia) in infants and toddlers.^1^, ^2^, ^3^ Prior to the implementation of universal RSV prophylaxis for young infants, RSV was estimated to cause globally 6.6 new million episodes of acute LRTI each year in infants < 6 months, leading to 1.4 million hospitalizations and ∼ 45,000 deaths annually, accounting for 3.6% of all deaths in this age group.^4^ Over 97% of RSV-attributable deaths occur in low middle income countries (LMIC). Importantly, community based-studies have shown that a significant proportion of RSV-related deaths occur outside hospital settings, especially in regions with limited access to care.^5^ These data emphasizes the need to provide universal protection against RSV from birth and through early infancy.

Up until recently, the only strategy approved for RSV prevention was passive prophylaxis with palivizumab. Palivizumab is a neutralizing humanized monoclonal antibody (IgG_1_) that targets antigenic site II located in the RSV F (fusion) protein in both the pre-F and post-F conformations, and has a half-life of 28 days, requiring monthly intramuscular administration during the RSV season. Palivizumab was approved by the FDA in 1998 for prevention of severe RSV disease in preterm infants and children with chronic lung disease (CLD), and subsequently in 2003, in those with congenital heart diseases (CHD).^6^^,^^7^ Since then, palivizumab has been implemented in more than 60 countries. Both placebo-controlled and observational comparative studies have consistently demonstrated its efficacy at preventing RSV-hospitalizations in these high-risk children, as well as an excellent safety record.^8^ Nonetheless, due to palivizumab high costs and the need for monthly administration studies have reported different challenges in the implementation and adherence to palivizumab prophylaxis in high risk-children.^9^

The study by Feitosa and Vieira published in this issue of the journal^10^ provides clear evidence that access and adherence to palivizumab in Brazil, remain major challenges. Authors conducted a retrospective study including ∼ 900 infants that had received palivizumab between 2008 and 2019 at the Vaccine and Immunobiological Unit in the Child and Adolescent Institute as part of the Medical School of Sao Paulo University. Their main goal was to analyze adherence to palivizumab prophylaxis and the barriers to its implementation. They found that fewer than half (44.5%) of eligible infants were completely adherent to the recommended prophylaxis regimen with palivizumab, defined as receiving the correct number of doses at the appropriate intervals. Specifically, geographic barriers and maternal sociodemographic factors — particularly younger maternal age — were associated with poor adherence, while infants with extreme prematurity and very low birth weight demonstrated somewhat better compliance. These findings underscore that structural and social determinants of health weigh as heavily as clinical eligibility in determining outcomes. In addition, the study did not find a clear association between full adherence to palivizumab and bronchiolitis-related hospitalizations, raising the question of whether partial prophylaxis may still confer protection, as reported in studies using abbreviated palivizumab dosing schedules.^11^ Nonetheless, although some of the bronchiolitis-related hospitalizations may have been caused by RSV, the lack of RSV testing precludes confirmation, especially considering that palivizumab confers protection only against RSV, and has no activity against other respiratory viruses.

Altogether, the study by Feitosa and Vieira underscores that reliance on an effective, costly, multi-dose monthly regimen that required administration in centralized centers contributes to inequities that ultimately limits the impact of palivizumab administration high-risk children.

The timing of this study is particularly relevant. As mentioned above, while there are well-defined risk factors for severe RSV disease, the majority of children that are hospitalized for RSV LRTI have no identifiable risk factors, underscoring the critical need for universal RSV prevention.^12^, ^13^, ^14^ In the last decade the development of RSV preventive strategies has witnessed a marked resurgence, overcoming the setbacks of the earlier vaccine trials conducted in the mid-1960s’. On the one hand, the recognition of the impact of RSV disease globally, and on the other hand, the identification of key antigenic sites in the RSV fusion (F) protein in its pre-F conformation, have been major drivers of this notable resurgence.^15^ There are currently three products approved for the prevention of RSV LRTI in young infants in the United States: a maternal bivalent preF vaccine, and two extended half-life human monoclonal antibodies, nirsevimab, and clesrovimab.^16^, ^17^, ^18^ Nirsevimab is a highly potent pre-F specific mAb that targets antigenic site Ø, while clesrovimab targets antigenic site IV located in the preF and postF conformations, and has enhanced penetration into the nasal epithelial lining fluid, which is crucial for neutralizing RSV at the primary site of infection.^19^ Recent studies evaluating the impact of the maternal RSV vaccine and of nirsevimab in the real world have demonstrated effectiveness that exceeds that reported in the original phase-III clinical trials, reducing RSV-related hospitalizations in infants by 70%–90% during their first RSV season.^20^, ^21^, ^22^

Nirsevimab, and maternal RSV vaccination are now approved in Brazil and offer scalable and are superior alternatives to the monthly palivizumab regimen. These strategies address adherence challenges and broaden protection to all infants including those with high-risk factors. Yet, the potential of these strategies needs to be weighed against their cost and the capacity of national health systems to implement and integrate them equitably, challenges that are specially pronounced in LMIC.^23^^,^^24^ In the U.S, both nirsevimab and the maternal RSV vaccine were approved in the summer of 2023. However, data from regional and national surveillance systems during the 2023-2024 season showed overall low uptake: only 29% of infants were protected, with 19% receiving nirsevimab and 10% covered through maternal vaccination.^25^ When both interventions were offered coverage increased substantially to 70%–80%. Nonetheless, lower uptake was identified among infants born to non-Hispanic Black and Middle Eastern or North African mothers, underscoring that inequities persist even within high-income countries.^26^

The study by Feitosa and Vieira highlights that preventing RSV hospitalizations requires not only effective drugs but also ensuring that children have access to them. As these new preventive strategies are introduced in Brazil and beyond, countries should strive to integrate RSV prevention within frameworks guided by equity and sustainability. Cost-effectiveness analyses, as shown in the US or Canada,^27^ proactive maternal immunization campaigns, and integration of RSV long-acting monoclonal antibodies into national immunization schedules are key steps. Only then will we bridge the gap between scientific advances and their real-world impact. Every year 140 million infants are born worldwide. The introduction of single-dose long-acting monoclonal antibodies and maternal vaccines against RSV have been transformative; the priority now is to ensure that no infant is left behind.
